# Comparative Study Between Conservative Versus Surgical Management of Iatrogenic Pancreatic Injury During Radical Nephrectomy

**DOI:** 10.7759/cureus.85895

**Published:** 2025-06-13

**Authors:** Ayman Abdulmohaymen, Abd Elfattah Al Sheikh, Tamer A. Ali, Mohamed Rehan, Saed Khater, Esam A. Elnady, Maha M. Elzamek, Osama M. Ghoneimy, Mohamed F. Elebiary, Mohamed Hindawy, Ahmed M. Aydarous, Satyabrata Garanayka, Ahmed Youssef, Khaled Monazea, Abdoh Salem, Ahmed Lamey

**Affiliations:** 1 Department of Surgical Oncology, Faculty of Medicine, Al-Azhar University, Cairo, EGY; 2 Department of Urology, Faculty of Medicine, Al-Azhar University, Cairo, EGY; 3 Department of Urology, Faculty of Medicine, Al-Azhar University, Damietta, EGY; 4 Department of Urology, Al-Azhar University, Cairo, EGY; 5 Department of Urology, Thumbay University Hospital, Ajman, ARE; 6 Department of General Surgery, Faculty of Medicine, Al-Azhar University, Assiut, EGY; 7 Department of General Surgery, Faculty of Medicine, Al-Azhar University, Cairo, EGY; 8 Department of General Surgery, Faculty of Medicine, Kafr Elsheikh University, Kafr Elsheikh, EGY

**Keywords:** conservative management, pancreatic fistula, pancreatic injury, radical nephrectomy, surgical management

## Abstract

Background

Pancreatic injury is a rare but serious complication of radical nephrectomy. The best management strategy for this complication, either conservative or surgical, remains debated, with limited comparative data.

Objective

To compare conservative management (CM) (drain placement, nutritional support, or somatostatin analogs) with surgical management (SM) (direct pancreatic repair, surgical drainage procedures, or partial pancreatectomy) in terms of outcomes, hospital stay, and mortality.

Patients and methods

A retrospective cohort study was conducted, including 30 patients who underwent radical nephrectomy with intraoperative pancreatic injury from January 2014 to January 2024. Patients were divided into two groups: Group 1, the CM group, which had 16 patients who underwent percutaneous drainage, octreotide, or enteral nutrition, and group 2, the SM group, which had 14 patients who underwent pancreatic repair, resection, or internal drainage. Both groups were compared in terms of complications, hospital stay, and mortality.

Results

The study groups were comparable regarding baseline patient criteria. Postoperative pancreatic fistula (POPF) was markedly less common in group 2 (14.3%; 2/14) than group 1 (37.5%; 6/16) (p=0.045). Mortality rates did not significantly differ between the trial arms. However, secondary outcomes revealed statistically significant differences between study groups in terms of hospital stay and failure/reintervention rates.

Conclusion

Surgical repair reduces POPF incidence and hospital stay, but CM is effective in minor injuries. A risk-based approach is recommended.

## Introduction

Nephrectomy is a significant urologic operation that has become more prevalent in recent decades [1]. It can be performed for a number of indications, but the most common ones are renal injuries, acute and chronic infections, non-functioning kidneys, donor nephrectomy for renal transplantation, and various renal cancers, primarily renal cell carcinoma (RCC) [2]. About 20% of patients have postnephrectomy complications. Common postoperative consequences include superficial and deep infections, renal failure, iatrogenic injury to any gastrointestinal organ, and bleeding, primarily from blood vessels close to the resected kidney or the renal pedicle. There are not many instances of iatrogenic injury to organs around the kidneys in the literature, making them uncommon consequences [3]. Only a small number of cases have been reported of pancreatic injuries during nephrectomy, making them much less common [4-6]. In contrast to splenic injuries, which are typically identified during surgery and treated with a splenectomy, pancreatic injuries are usually identified after surgery when pancreatic fluid collections and/or fistulas become clinically noticeable. Furthermore, it has been demonstrated that patients with iatrogenic pancreatic injuries have considerable fatalities and may need surgical reintervention [7]. The International Study Group of Pancreatic Surgery (ISGPS) created a widely recognized definition and classification system for postoperative pancreatic fistula (POPF) in 2005 [8]. The ISGPS fistula classification has emerged as the gold standard for defining POPF in clinical practice since it is still one of the most significant and detrimental complications. In 2016, this grading system was updated in view of newly discovered data and to resolve the ongoing disputes regarding the initial classification and definition of POPF [9]. Risk factors for POPF after radical nephrectomy include large tumor size, extracapsular extension, metastatic lymph nodes, postneoadjuvant cases, and poor patient general condition. All these factors share the need for extra dissection or patient fragility. Our objective in this study was to clarify this significant issue and the potential treatment options [6].

## Materials and methods

This retrospective multicenter study investigated a ten-year period between January 2014 and January 2024. Patients were included in this study if they had a radical nephrectomy in this time interval and sustained iatrogenic pancreatic injury. However, exclusion criteria were pre-existing pancreatic disease and metastatic RCC requiring systemic therapy. Iatrogenic pancreatic injury is defined as unintended pancreatic trauma during surgery, driven by anatomical vulnerability and technical factors. Its hallmark is enzymatic leakage, leading to fistulas or collections, with diagnosis relying on biochemical/imaging criteria. A clear diagnosis was made by operative drains having an output of more than 50 mL/day, with the drain amylase level more than three times its serum level. Pre-existing pancreatic disease refers to any structural, inflammatory, neoplastic, or functional disorder of the pancreas diagnosed or present before surgery that may alter pancreatic anatomy (e.g., fibrosis, duct dilation, and parenchymal atrophy), increase vulnerability to iatrogenic injury (e.g., inflammation-induced tissue friability), or impair healing or amplify complications (e.g., reduced regenerative capacity and predisposition to fistulas). Thirty cases were obtained from three surgical oncology centers. Patients were grouped into two treatment groups (study arms) according to ISGPS grading criteria [9]. Group 1 (conservative management or CM group) included 16 patients who had percutaneous drainage (>50 mL/day), somatostatin analogs (octreotide 100 mcg three times a day (TID), continued until the output was less than 50 mL/day), and low-fat, medium-chain triglyceride (MCT)-based enteral diet. Total parenteral nutrition and antibiotics were initiated in four cases, with clinical and laboratory evidence of sepsis and no response to the above regimen. Unfortunately, these four patients failed to respond to CG and needed surgical intervention. Initially, operative drains were used to monitor the daily output amount and amylase content. When daily ultrasound follow-up after initial diagnosis of iatrogenic pancreatic injury revealed undrained collections, percutaneous drains were placed. Therefore, the criteria for conservative treatment were completely drainable discharge with no residual collection, no clinical or laboratory signs of sepsis, and low output of less than 500 mL/day. However, any change in this criterion or persistence of output more than 50 mL/day after four weeks was considered a failure of CM requiring surgical intervention. Group 2 (surgical management or SM group) included 14 patients who underwent surgical procedures that varied from primary repair (pancreatic suturing, glue, and mesh) to external drainage (pancreatic cyst or fistula drainage). However, four cases required pancreatic resection (distal pancreatectomy). Criteria for surgical intervention included undrainable discharge or residual collection, clinical or laboratory signs of sepsis, and high output of more than 500 mL/day. Persistence of any of these criteria on the seventh day after surgical intervention was considered a failure of surgical intervention requiring resurgery.

Using the following diagnosis codes (acute pancreatitis, pancreatic fistula, and pancreatic damage), complications and hospitalization data were retrieved from the medical records. Every intraoperative, postoperative, and hospitalization-related issue that arises in the first postoperative month was recorded. Both study groups were compared regarding the primary outcome, incidence of pancreatic fistula (POPF), secondary outcomes, reintervention rates, and length of hospital stay. Finally, both groups were compared in terms of one-year mortality. The primary endpoint of our study was the resolution of the complication with drain output less than 50 mL/day. Secondary endpoints were at six months and one-year follow-up.

Ethical consideration

Every procedure used in research involving human subjects complied with the national and institutional research committee's ethical guidelines. Written informed consents for the treatment and future publication were obtained from all patients before giving any treatment modality. 

Statistical analysis

We analyzed the data using the Statistical Package for Social Science (SPSS), version 29 (IBM Corp., Armonk, NY). The descriptive statistics included percentages, frequencies, means, and medians. We compared categorical variables between the two groups using the Chi-square test. Additionally, for hospital stay, we used the student’s t-test. Statistical significance was defined as a two-tailed p-value of less than 0.05.

## Results

The 10-year span from January 2014 to January 2024 was examined in this retrospective multicenter study. In this time period, out of 712 patients having radical nephrectomy, 30 patients had pancreatic injury, representing a 4.2% overall incidence. These patients with pancreatic injuries were divided into two study groups: group 1, the CM group, and group 2, the SM group. Group 1 (CM group) included 16 patients. This group received conservative treatment through percutaneous drainage, somatostatin analogs (octreotide 100 mcg TID), and a low-fat, MCT-based enteral diet. Additionally, antibiotics and total parenteral nutrition were given in four patients when infection was suspected clinically and confirmed by laboratory testing. Later on, these four patients failed to improve and needed surgical intervention. Out of them, one patient developed septic shock, was admitted to the ICU, and then died. Group 2 (SM group) received variable procedures. While six cases underwent primary repair, four other patients needed external drainage, and four patients needed major intervention in the form of distal pancreatectomy. There were no statistically significant differences between study groups regarding demographic criteria: age and sex, with the mean age in the CM group being 56±10 years compared to 54±9 years in the SM group (Table 1).

**Table 1:** Patient demographics CM, conservative management; SM, surgical management

Item	CM group	SM group	p-value
Age	56±10 years	54±9 years	0.5
Sex	10 females, 6 males	8 females, 6 males	1

POPF rate showed a statistically significant difference between study arms, as it was markedly less common in the SM group (14.3%; 2/14) when compared to the CM group (37.5%; 6/16) (p=0.045). Our study outcomes showed statistically significant differences between study groups regarding hospital stay and failure/reintervention rates, while mortality rates had no significant difference between the study arms (Table 2).

**Table 2:** Secondary outcomes CM, conservative management; SM, surgical management

Item	CM group	SM group	p-value
Hospital stay	22±3 days	16±2 days	0.04
Failure/reintervention rates	4 cases (25%)	2 cases of reoperation (14%)	0.03
Mortality (6 and 12 months)	1 case	0	0.47

## Discussion

According to our results, CM is effective for low-grade injuries but fails in 25% of cases, while SM reduces fistula risk and hospital stay but requires expert surgical intervention. Significant morbidity and mortality are associated with iatrogenic pancreatic injury. Clinical suspicion is the basis for diagnosis, which is supported by radiologic and laboratory evidence, such as amylase in the drained fluid. Nothing by mouth (NPO), fluid resuscitation, percutaneous drainage of the pancreatic fluid, and antibiotic treatment if infection is suspected are all components of CM [9]. Surgery is necessary for about 10% of patients with pancreatic injuries, and success rates are 90%-92%; nevertheless, there is a 6%-9% higher risk of death with these procedures [10]. The majority of pancreatic iatrogenic injuries are not identified during surgery; instead, they are discovered clinically after the procedure. The availability of fluid for amylase testing facilitates the diagnosis in cases where a drain was left in place after surgery. Amylases are measured to confirm diagnosis, and they are often high. Clinically severe pancreatic fistula is linked to an increased amylase in the drain of 2820 U/L and higher [11]. Treatment for a pancreatic leak should be determined by the clinical state of the patient. Re-evaluation should be performed every few days if the leak is contained within the drain. An NPO order should be used to reduce pancreatic secretion if the postoperative symptoms are associated with pancreatic injury or if the leak happens in considerable amounts (more than 50 mL/day) [12]. Four patients with pancreatic damage were reported out of 890 patients who had laparoscopic upper urinary tract procedures in Varkarakis et al.'s largest study to date [13]. This represents an incidence rate of 0.44%. Liapis and colleagues [6] reviewed 600 patients with retroperitoneal urologic surgeries 10 years ago. Out of these, 32 (5.3%) experienced POPF. A case of significant pancreatic fluid accumulation over two months after nephrectomy, which was effectively treated with percutaneous drainage, is described in a recent case report by Bozkurt et al. [5]. The management of pancreatic injury in relation to various kidney surgeries, including the extent of surgery (partial, radical, and simple nephrectomy), was not investigated in any of these papers. The findings of these reports and ours indicate that most patients with pancreatic injuries after urologic interventions can be treated without surgical intervention, provided that effective drainage with the interventions is achieved. However, when the initial procedure does not involve an attempt to manipulate the pancreas, surgical intervention appears to be more common in iatrogenic pancreatic injuries. This could be because postoperative clinical suspicion is low, as these injuries are uncommon. Inadequate drainage may also be associated with it. Patients with large T3 and T4 tumors who had radical nephrectomy for large renal tumors have a greater risk of iatrogenic pancreatic injury [14]. Even though pancreatic injury is very uncommon after radical nephrectomy, these patients are at risk for a prolonged hospital stay, as well as percutaneous and surgical procedures that have a high rate of postoperative morbidity. The prolonged duration of stay after pancreatic iatrogenic injury in our study demonstrates the substantial impact of this complication. Our study has some limitations, being retrospective with a relatively small sample size. This can be explained by the rare incidence of the reported complication during radical nephrectomy. However, the large number of cases (712 cases) that underwent radical nephrectomy and were originally examined to include such a sample can mitigate the effect of these limitations.

## Conclusions

Our study concluded that CM is effective in most cases presented with minor pancreatic injuries but fails in the minority of instances having high-grade leaks. On the other hand, SM reduces POPF and hospital stay and is recommended for major injuries. A risk-stratified approach is needed for individualized patient management.

## References

[1] Touijer K, Jacqmin D, Kavoussi LR (2010). The expanding role of partial nephrectomy: a critical analysis of indications, results, and complications. Eur Urol.

[2] Schiff M Jr, Glazier WB (1977). Nephrectomy: indications and complications in 347 patients. J Urol.

[3] Tan K, Lewis GR, Chahal R (2011). Iatrogenic splenectomy during left nephrectomy: a single-institution experience of eight years. Urol Int.

[4] Siebels M, Theodorakis J, Schmeller N (2003). Risks and complications in 160 living kidney donors who underwent nephroureterectomy. Nephrol Dial Transplant.

[5] Bozkurt M, Can O, Altunrende F (2017). A pancreatic fistula as a rare complication of laparascopic radical nephrectomy: a case report. Urol Case Rep.

[6] Liapis D, de la Taille A, Ploussard G (2008). Analysis of complications from 600 retroperitoneoscopic procedures of the upper urinary tract during the last 10 years. World J Urol.

[7] Sanjay P, Kellner M, Tait IS (2012). The role of interventional radiology in the management of surgical complications after pancreatoduodenectomy. HPB (Oxford).

[8] Bassi C, Marchegiani G, Dervenis C (2017). The 2016 update of the international study group (ISGPS) definition and grading of postoperative pancreatic fistula: 11 years after. Surgery.

[9] Bassi C, Dervenis C, Butturini G (2005). Postoperative pancreatic fistula: an international study group (ISGPF) definition. Surgery.

[10] Alexakis N, Sutton R, Neoptolemos JP (2004). Surgical treatment of pancreatic fistula. Dig Surg.

[11] Ceroni M, Galindo J, Guerra JF, Salinas J, Martínez J, Jarufe N (2014). Amylase level in drains after pancreatoduodenectomy as a predictor of clinically significant pancreatic fistula. Pancreas.

[12] Chang YR, Kang MJ, Kim H, Jang JY, Kim SW (2016). The natural course of pancreatic fistula and fluid collection after distal pancreatectomy: is drain insertion needed?. Ann Surg Treat Res.

[13] Varkarakis IM, Allaf ME, Bhayani SB, Inagaki T, Su LM, Kavoussi LR, Jarrett TW (2004). Pancreatic injuries during laparoscopic urologic surgery. Urology.

[14] Lerut J, Lerut T, Gruwez JA, Michielsen P, Van Renterghem I (1980). Surgical gastro-intestinal complications in 277 renal transplantations. Acta Chir Belg.

